# Viral species richness and composition in young children with loose or watery stool in Ethiopia

**DOI:** 10.1186/s12879-019-3674-3

**Published:** 2019-01-14

**Authors:** Kristen Aiemjoy, Eda Altan, Solomon Aragie, Dionna M. Fry, Tung G. Phan, Xutao Deng, Melsew Chanyalew, Zerihun Tadesse, E. Kelly Callahan, Eric Delwart, Jeremy D. Keenan

**Affiliations:** 1 Francis I. Proctor Foundation, University of California San Francisco, 513 Parnassus Avenue, MedSci S309, Box 0412, San Francisco, CA 94143 USA; 2 Department of Epidemiology and Biostatistics, University of California San Francisco, San Francisco, USA; 3 Blood Systems Research Institute, San Francisco, USA; 4 Department of Laboratory Medicine, University of California San Francisco, San Francisco, USA; 5 Amhara Regional Health Bureau, Bahir Dar, Ethiopia; 6 The Carter Center Ethiopia, Addis Ababa, Ethiopia; 7 The Carter Center, Atlanta, GA USA; 8 Department of Ophthalmology, University of California San Francisco, San Francisco, USA

**Keywords:** Diarrheal disease, Virome, Viral infection, Norwalk virus

## Abstract

**Background:**

Stool consistency is an important diagnostic criterion in both research and clinical medicine and is often used to define diarrheal disease.

**Methods:**

We examine the pediatric enteric virome across stool consistencies to evaluate differences in richness and community composition using fecal samples collected from children aged 0 to 5 years participating in a clinical trial in the Amhara region of Ethiopia. The consistency of each sample was graded according to the modified Bristol Stool Form Scale for children (mBSFS-C) before a portion of stool was preserved for viral metagenomic analysis. Stool samples were grouped into 29 pools according to stool consistency type. Differential abundance was determined using negative-binomial modeling.

**Results:**

Of 446 censused children who were eligible to participate, 317 presented for the study visit examination and 269 provided stool samples. The median age of children with stool samples was 36 months. Species richness was highest in watery-consistency stool and decreased as stool consistency became firmer (Spearman’s *r* = − 0.45, *p* = 0.013). The greatest differential abundance comparing loose or watery to formed stool was for norovirus GII (7.64, 95% CI 5.8, 9.5) followed by aichivirus A (5.93, 95% CI 4.0, 7.89) and adeno-associated virus 2 (5.81, 95%CI 3.9, 7.7).

**Conclusions:**

In conclusion, we documented a difference in pediatric enteric viromes according to mBSFS-C stool consistency category, both in species richness and composition.

**Electronic supplementary material:**

The online version of this article (10.1186/s12879-019-3674-3) contains supplementary material, which is available to authorized users.

## Background

Stool consistency is an important diagnostic criterion in both research and clinical medicine [1]. Changes in stool consistency are used to measure many gastrointestinal disorders such as ulcerative colitis, irritable bowel syndrome and diarrhea [2–6]. Most epidemiologic studies of diarrheal disease internationally use stool consistency, specifically ‘loose or watery stool’ to classify diarrhea cases [2, 7].

Visual and descriptive stool consistency scales may standardize and improve the accuracy of reported stool consistency. The most widely used stool form scale, The Bristol Stool Form Scale (BSFS), was developed in the late 1980s to measure gut transit time [8, 9]. The BSFS classifies stool form into seven categories according to stool cohesion, surface cracking and consistency.

The BSFS was later simplified to a five-level scale and renamed the modified Bristol Stool Form Scale for children (mBSFS-C) [10], ranging from type 1 (hard pellets) to type 5 (watery stool).

While recent studies have described the bacterial microbiome of the colon and feces, there have been few parallel investigations of the enteric and stool virome [11–14]. Healthy gut and fecal bacterial microbiomes are characterized as having higher species richness [15]. This relationship may not hold for the fecal virome, where higher species richness may signal more viral infections and disease.

Here we use metagenomics to examine the pediatric fecal virome across standardized stool consistency categories using stool samples from 269 children aged 0 to 5 years in rural Ethiopia. We evaluate potential associations between enteric virome composition, species richness and stool consistency.

## Methods

### Study design

This study was conducted during the final annual visit of a clinical trial evaluating a water improvement intervention in the Amhara region of Ethiopia (clinicaltrials.gov NCT02373657) [16–18]. Methods for the parent trial are described in detail elsewhere [19, 20]. A door-to-door population census was taken in all communities before the study visit. All children aged 0–5 years enumerated on the census were eligible to participate in the study and provide stool samples. The final study visit occurred in April 2016; April is the dry season in this region. The study visit is held in a central location in the community.

### Stool sample collection and grading

Caregivers were instructed to have their child defecate in a plastic child’s potty chair lined with a black plastic bag. For children unable to produce a stool in the field, supplies were provided to the caregiver, with instructions to collect stool at home the following morning and bring it to a collection site the following day at a designated time. Stool samples that were collected at home were stored at room temperature.

When the stool was returned to the field station, it was independently inspected in the original collection container and graded according to the Modified Bristol Stool Form Scale for Children (mBSFS-C) by two trained laboratory technicians. Laboratory technicians underwent a two-day training on sample collection and consistency grading prior to data collection. The first laboratory technician’s grade was used to classify stool consistency for this analysis.

**Fig. 1:**
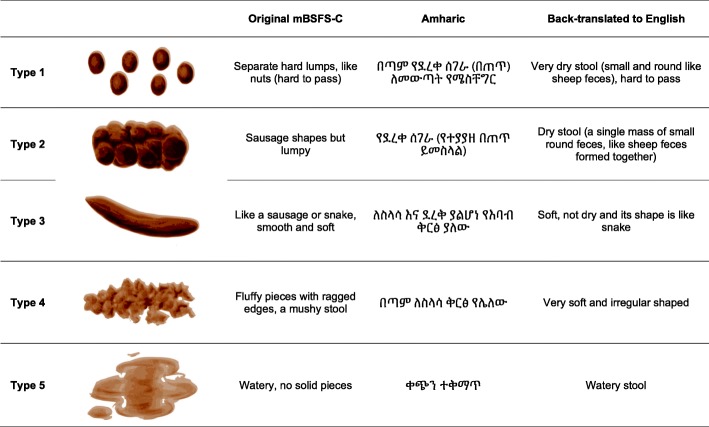
The modified Bristol Stool Form Scale for Children (mBSFS-C), translated into Amharic

The mSFS-C was available as a laminated sheet with both the cartoon images and Amharic translations (Fig. 1). Methods describing the grading process and kappa evaluating agreement are described in detail elsewhere [21].

After the stool sample consistency was graded, 0.5 mL of stool was placed in a 1 mL plastic tube, put on ice and transferred to a − 20 C freezer at the end of the day. At the completion of the sample collection, in early May 2016, all samples were transferred to Bahir Dar Regional Laboratory (Bahir Dar, Ethiopia) and kept at − 20 C until they were shipped to University of California, San Francisco in February 2017.

### Laboratory methods

Stool specimens were combined into pools of 6 to 12, with sampling stratified by stool consistency grade. Pools were clarified using a tabletop microfuge at 15,000 rpm for ten minutes. Supernatants were then filtered using a 0.45-μm filter (Millipore). The filtrates were digested with a cocktail of nuclease enzymes. Viral nucleic acids protected from digestion within their protein capsids were then extracted using the automated extractor Maxwell 16 (Promega) [22]. Random RT-PCR was followed by the use of the Nextera™ XT Sample Preparation Kit (Illumina) to generate libraries for Illumina MiSeq (2 × 250 bases) using dual barcoding as described [23, 24].

### Bioinformatic analyses

Human, bacterial, duplicate, and low-quality sequence reads were first removed. The remaining reads were then de novo assembled. BLASTx translated contigs and singlet reads were aligned against a customized viral proteome database. Candidate viral hit sequences were then aligned against a non-redundant non-virus protein database. Those candidates yielding E score higher (weaker) than those against viral proteins were removed to decrease false positive viral hits [23, 24]. A detailed description of the bioinformatics pipeline is available in the supplemental material (Additional file 1). 

### Statistical methods

All statistical analyses were performed in R version 3.4.2 (R Foundation for Statistical Computing, Vienna, Austria) using R Studio version 1.1.383. In the interest of reproducibility, an R markdown document containing complete commands for the analysis is available here: https://github.com/kaiemjoy/fecalvirome. The number of viral reads along with the taxonomic assignments and sample characteristics were assembled using the phyloseq package. We define absolute abundance as the raw number of reads for each species/genotype and relative abundance as the proportion of raw reads of each species/genotype in each pool. To calculate relative abundance, we divided the number or reads for each species/genotype in each pool by the total number of reads in that pool and multiplied by 100. We evaluated species richness (Observed, Chao1) and alpha diversity measures (Simpson, Shannon, and Fisher) using the *estimate_richness* function of phyloseq. We used Spearman’s rank order test to assess correlation between richness and mBSFS-C stool consistency category and determine statistical significance.

We determined differential abundance comparing loose and watery (mBSFS-C types 4&5) to formed stool (mBSFS-C types 1–3) at the species/genotype level using negative-binomial modeling in the *DESeq2* package [25]. We used the Bonferroni method to adjust *p*-values for multiple comparisons. We explored both an unadjusted model and a model adjusted for age. To adjust for age, we calculated the median age for each pool and included it as a covariate in the model. Results are expressed as log_2_ fold change in loose or watery stool compared to formed stool.

### Ethics statement

Ethical committees at the University of California (San Francisco, CA, USA); Emory University (Atlanta, GA, USA); The Food, Medicine and Health Care Administration and Control Authority of Ethiopia; and the Ethiopian Ministry of Science and Technology granted approval for this study. We obtained verbal informed consent in Amharic from all caregivers.

## Results

### Characteristics of study population

Of 446 censured children who were eligible to participate, 317 children presented for the study visit examination and 269 provided stool samples. The median age of children with stool samples was 36 months (IQR 12–48), 53.2% (143/269) of children were female. A detailed description of the study population characteristics by stool consistency category is presented in Table 1. The 269 samples were analyzed in 29 pools: 4 pools (29 samples) were watery/type 5, 8 pools (79 samples) were loose/type 4, 6 pools (59 samples) were smooth/type 3, 9 pools (88 samples) were lumpy/type 2 and 2 pools (12 samples) were hard pellets/type1. The median age for children with watery/type 5 stool was 7.2 months, younger than with loose/type 4 stool (36 months), smooth/type 3 (36 months), lumpy/type 2 (36 months) or pellet/type 1 (48 months). Caregivers reported observing blood in the stool during the past seven days in 4/29 (13.8%) of children with watery stool samples, 4/79 (5.1%) of children with loose stool samples, 2/59 (3.4%) of children with smooth stool samples, 5/88 (5.7%) of children with lumpy stool samples and 0/14 (0%) of children with pellet stool samples.

**Table 1:** Characteristics of the study population by mBSFS-C stool consistency category

N children (pools)	Watery 29 (4)	Loose 79 (8)	Smooth 59 (6)	Lumpy 88 (9)	Pellets 14 (2)
Median age in months (IQR)	7.2 (4.8-12)	36 (12-48)	36 (24-48)	36 (24-49.2)	48 (36-57)
Female	15 (51.7% )	39 (49.4% )	33 (55.9% )	48 (54.5% )	8 (57.1% )
Blood in stool^a^	4 (13.8% )	4 (5.1% )	2 (3.4% )	5 (5.7% )	0 (0% )
Fever^b^	9 (32.1% )	28 (36.8% )	20 (35.1% )	33 (38.8% )	6 (50% )

Numbers are N (%) unless otherwise indicated

^a^Caregiver reported observing blood in the stool any day in the past seven days

^b^Caregiver reported child had a fever any day in the past seven days

### Prevalence

**Fig. 2:**
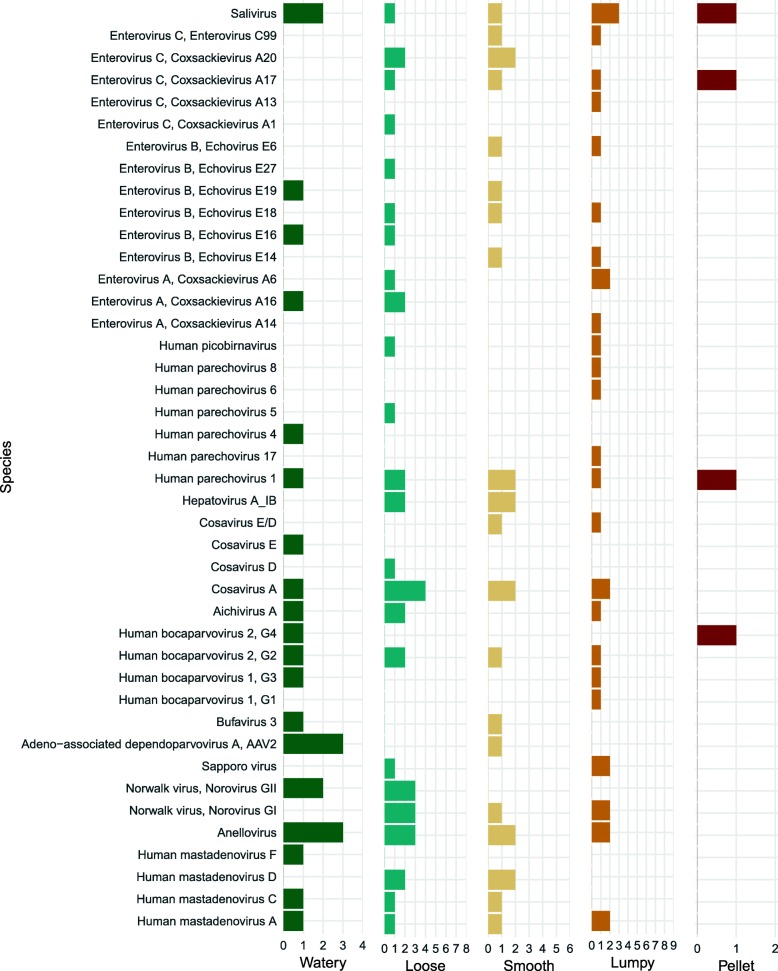
Prevalence of species/genotypes by mBSFS-C stool consistency category

The most prevalent viral reads belonged to anelloviruses (10 of 29 pools), picornaviruses in the species cosavirus A (9 of 29 pools), and salivirus A (8 of 29 pools). Several viruses/genotypes were more prevalent in watery or loose stool compared to formed stool (Fig. 2). For example, Norovirus GII was detected in 5 of 12 (42%) loose or watery pools and 0 of 17 formed pools. Aichivirus A was detected in 3 of 12 (25%) loose or watery pools and 1 of 17 (6%) formed pools. Human mastadenovirus C was detected in 2 of 12 (17%) loose or watery pools and 1 of 17 (6%) formed pools. For some viruses, such as salivirus, Human parechovirus 1, and Saporro virus, there did not appear to be a difference in prevalence by stool consistency category. Human mastadenovirus D was only detected in 1 of 4 (25%) watery pools and no other pools. Rotavirus was not detected in any samples (Additional file 2: Table S1).

### Abundance

**Fig. 3:**
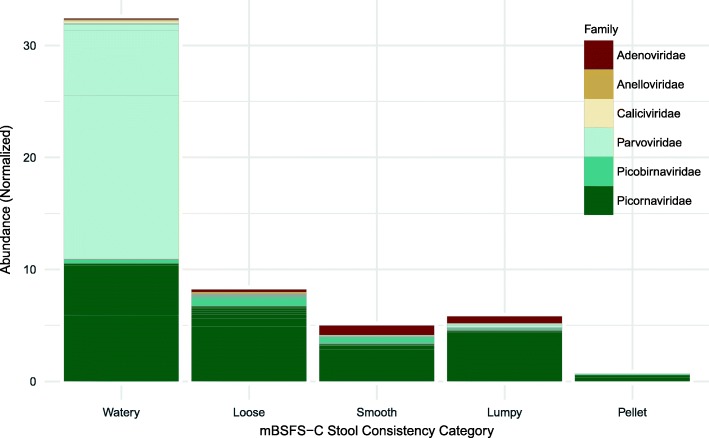
Viral abundance by mBSFS-C stool consistency category

The most abundant reads belonged to the family *Picornaviridae* followed by *Parvoviridae*. Both *Picornaviridae* and *Parvoviridae* reads had the highest abundance in watery-consistency pools, followed by loose, smooth and lumpy consistency with the lowest abundance in pellet-consistency pools (Fig. 3).

**Fig. 4:**
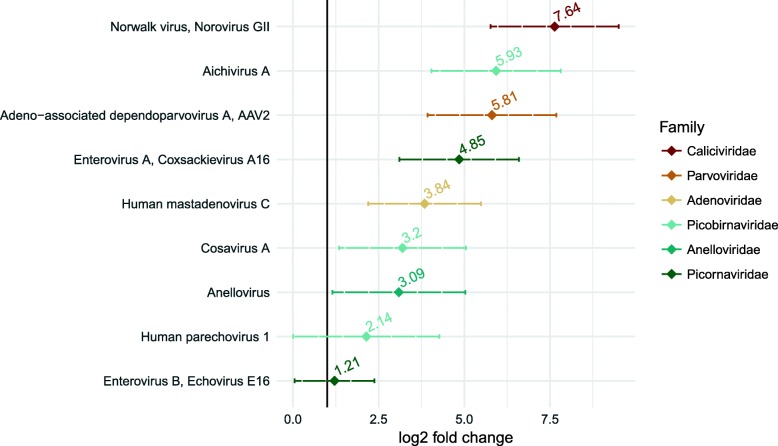
Differential abundance: Loose/watery stool compared to formed stool

The most abundant species/genotype, both in terms of absolute and relative abundance, was adeno-associated dependoparvovirus A, adeno-associated virus 2 (AAV2) followed by saliviruses, human bocavirus 2, and echovirus E19. The greatest differential abundance comparing loose or watery stool to formed stool was for norovirus GII, followed by aichivirus A, AAV2, coxsackievirus A16, human mastadenovirus, cosavirus A and anellovirus (Fig. 4). For norovirus GII, the abundance was 2656 reads in loose in watery consistency pools and 0 reads in formed consistency with a differential abundance of 7.64 (95% CI 5.8–9.5, *p* = < 0.001) comparing loose or watery stool to formed stool. For aichivirus A, the abundance was 5659 reads in loose or watery stool compared to 86 reads in formed stool, with a differential abundance of 5.93 (95% CI 4.0–7.8, *p* = < 0.001). The abundance of AAV2 was 115,946 in loose or watery-consistency pools and 222 in formed-consistency pools with a differential abundance of 5.81 (95%CI 16.0–27.6, *p* = < 0.001) (Table 2).

**Table 2:** Absolute abundance and log_2_ fold change

Species	Abundance in loose stool	Abundance in formed stool	log_2_ fold change (95% CIs)	*p*-value	adjusted *p*-value*
Norwalk virus, Norovirus GII	2656 (0.26)	0 (0)	7.64 (5.8 , 9.5)	<0.001	<0.001
Aichivirus A	5659 (0.38)	86 (0.01)	5.93 (4.0 , 7.8)	<0.001	<0.001
Adeno-associated dependoparvovirus A, AAV2	115946 (14.73)	222 (0.03)	5.81 (3.9 , 7.7)	<0.001	<0.001
Enterovirus A, Coxsackievirus A16	2561 (0.22)	0 (0)	4.85 (3.1 , 6.6)	<0.001	<0.001
Human mastadenovirus C	3023 (0.27)	449 (0.03)	3.84 (2.2 , 5.5)	<0.001	<0.001
Cosavirus A	932 (0.09)	122 (0.02)	3.2 (1.3 , 5)	0.001	0.003
Anellovirus	2515 (0.28)	427 (0.06)	3.09 (1.2 , 5)	0.002	0.006
Parechovirus A, Human parechovirus 1	3545 (0.53)	1986 (0.24)	2.14 (0 , 4.3)	0.049	0.071
Enterovirus B, Echovirus E16	71447 (4.44)	0 (0)	1.21 (0 , 2.4)	0.040	0.064

*p-value adjusted for multiple comparisons using the Bonferroni method

### Richness and alpha diversity

**Fig. 5:**
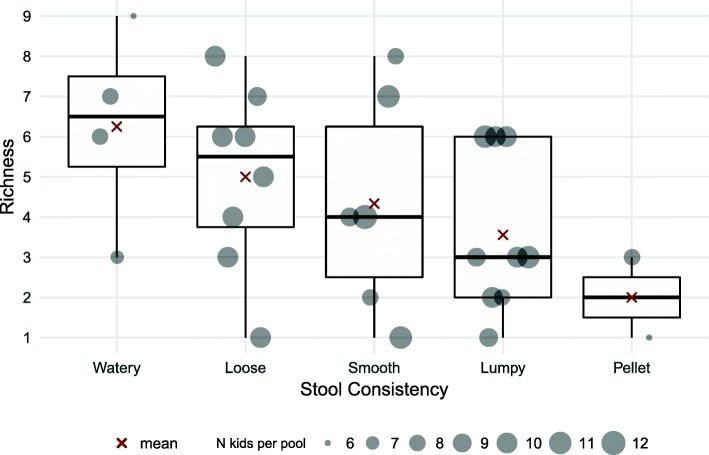
Species richness according to mBSFS-C stool consistency category

Species richness was highest in watery-consistency stool and decreased consistently as stool consistency became firmer (Spearman’s *r* = − 0.45, *p* = 0.013). The median number of distinct viruses was 6.5 (IQR 2.25) for watery stool, 5.5 (IQR 2.5) for loose stool, 4.0 (IQR 3.75) for smooth-consistency stool, 3.0 (IQR 4) for lumpy-consistency stool and 2.0 (IQR 1) for pellet-consistency stool (Fig. 5).

There was no association between stool consistency and Simpson alpha diversity (Spearman’s *r* = − 0.05, *p* = 0.80) or Shannon alpha diversity (Spearman’s *r* = − 0.08, *p* = 0.67). With the Fisher alpha diversity metric, loose and watery stool appeared to be more diverse than formed stool, but the difference was not statistically significant (Spearman’s *r* = − 22, *p* = 0.238) (Additional file 3: Figure S1).

## Discussion

We document differences in the richness and composition of the pediatric enteric virome by stool consistency. There was a marked and statistically significant decrease in the number of distinct virus species as stool consistency became firmer, with the highest number of distinct species in watery (mBSFS-C type 5) stool and the lowest number of distinct species in firm pellet stool (mBSFS-C type 1). Loose and watery stool types were more likely to have norovirus GII, aichivirus A, adeno-associated dependoparvovirus A - AAV2, coxsackievirus A16, human mastadenovirus C, cosavirus A and annelovirus compared to formed stool.

Our findings regarding species richness contrast with many studies in the bacterial microbiome literature which report elevated species richness in healthy individuals and stool. For example, a study of the fecal bacterial microbiome according to self-reported BSFS stool consistency and found that women with looser stool had fewer distinct bacterial species compared to women with firmer stool [13]. Virus richness, unlike bacterial richness, may be a marker for disease rather than health. Stool consistency is often used in symptomatic definitions of diarrhea, in particular, the World Health Organization definition (“three or more loose or watery stools in a 24 h period [2]”). Our findings of elevated species richness in watery and loose stool support the content validity of consistency-based definitions as they relate to infectious episodes of diarrhea.

Noroviruses are the leading cause of epidemic viral gastroenteritis globally [26]. We detected norovirus genotype II and genotype I in a fifth of pools, with genotype II showing the highest differential abundance in loose or watery stools compared to formed stools. Norovirus genotype II has been shown to be elevated in children with diarrhea and or gastroenteritis in many regions, including Africa [27, 28].

The second highest differential abundance we detected was for aichivirus A, which was detected 25% of loose or watery pools and 6% of formed pools. Aichivirus A has been detected in stool samples from children with diarrhea in France [29], Brazil [30], Tunisia [31] and several countries in Asia [32, 33]. We likewise found an elevated abundance of adeno-associated dependoparvovirus A - AAV2 in loose or watery consistency pools compared to formed consistency pools. AAV2 needs a helper virus, commonly an adenovirus, to infect the gastrointestinal tract and it is not thought to be pathogenic or cause diarrheal disease [34]. However, adeno-associated viruses have been detected in children with diarrhea using metagenomic sequencing [35]. Indeed, we detected adenovirus in three of the four pools with detected AAV2 reads.

We also found an increased abundance of coxsackievirus A16 in loose or watery consistency pools compared to formed pools. Coxsackievirus A16 is a leading cause of hand foot and mouth disease. Hand-foot and mouth infections are common in young children and we suspect that age may confound the association between stool consistency and coxsackie A16 abundance. In the median age-adjusted model, the differential abundance of coxsackie A16 in loose or watery stools compared to formed stools decreased to 1.78, and the 95% confidence intervals included the null effect of 1.0.

Enteric human adenoviruses, primarily human mastadenovirus F, are considered the third leading cause of nonbacterial diarrhea globally [27, 36]. We detected human mastadenovirus F in just one watery pool with too few reads to detect a statistically significant difference. We did detect a signal for higher differential abundance of human mastadenovirus C in loose or watery stool compared to formed stool although the *p*-value was not statistically significant. Studies in Albania, Korea and Asia have all detected human mastadenovirus C in children with diarrhea and gastroenteritis, but these studies lacked healthy controls to compare prevalence [37–39]. A study of human adenovirus in diarrhea children in Tanzania did not find a difference in the prevalence of human mastadenovirus F between children with and without diarrhea but did report a human mastadenovirus C prevalence of 12.5% in diarrhea cases and 7.7% in controls (prevalence ratio of 1.6) [40]. Human mastadenovirus C may be an unrecognized cause of pediatric diarrhea in Africa and warrants additional research with a larger sample size.

Cosavirus A was first identified 2008 and has since been recognized as a cause of diarrhea in children [41–44]. In our sample, Cosavirus A was 3.2 times more abundant in loose or watery stool compared to formed stool. Additional un-pooled studies would help confirm if Cosavirus A may be a cause of loose or watery stools in this population. Annelovirus is a nearly ubiquitous virus in human blood and its presence in the pooled samples may indicate blood in the feces, particularly in loose and watery stool samples [45, 46]. We did not detect any rotavirus, a leading cause of pediatric diarrhea. Ethiopia initiated a country-wide vaccination campaign in November of 2013, reaching a coverage of 85% by 2015 [47]. Successful rotavirus vaccination may explain why no rotavirus was detected in loose, watery or formed stool.

There are several limitations of this study that are important to consider. Our samples were pooled, reducing the effective sample size from 269 individual stool specimens to 29 pools. With a larger sample size (smaller pools) or individually run samples, we would have had more statistical precision for evaluating differences in the fecal virome by measured stool consistency. However, pooling has been shown to be an efficient strategy to accurately estimate prevalence when resources are limited [48–50]. The kappa measuring agreement between the laboratory technicians stool consistency grades was .72, introducing some possible misclassification of stool consistency into our analyses. This misclassification, likely non-differential, would on average bias any associations towards the null. Laboratory staff were not masked to the consistency of the pools when running the analyses. However, the samples were processed in an arbitrary order, not according to stool consistency. This study was conducted at the final study visit of a trial evaluating the effect of a water-improvement intervention on ocular chlamydia. However, we did not find any measurable effect of the water intervention on the enteric virome, as reported in a companion paper [51]. We did not run any of the pools in duplicate to assess repeatability. Another potential limitation is selection bias as only 269 of the 317 eligible children were able to provide a stool sample. However, we found no statistically significant difference in the age or gender of children who provided stool samples compared to those who did not.

Finally, fecal specimens were stored without media, although this should not affect identification of viruses as it would for bacteria.

Despite these limitations, our study had several strengths. Unlike many comparable studies using targeted PCR to detect specific pre-specified viruses, we used a metagenomic approach allowing systematic and unbiased characterization of the stool virome. We applied modern statistical tools developed for microbiome analysis, enabling a comprehensive view of the entire fecal viral community. Our study was also population-based, enrolling a representative sample of all children in the community, both healthy and sick.

## Conclusions

We found differences in the pediatric fecal virome by stool sample consistency as measured by the modified Bristol Stool Form Scale for Children (mBSFS-C), both in terms of species richness and composition. Watery and loose stools had greater species richness compared to formed stools and were more abundant in norovirus GII, aichivirus A, AAV2 and human mastadenovirus C.

## Additional files

Additional file 1: Bioinformatics Pipeline. (PDF 29 kb)

Additional file 2: **Table S1.** Prevalence of species/genotype by mBSFS-C stool consistency category. (PDF 58 kb)

Additional file 3: **Figure S1.** Alpha Diversity according to mBSFS-C stool consistency category. (PDF 19 kb)

## Abbreviations

AAV2: Adeno-associated dependoparvovirus A
BSFS: Bristol Stool Form Scale
CI: Confidence interval
IQR: Inter-Quartile Range
mBSFS-C: Modified Bristol Stool Form Scale for Children
PCR: Polymerase Chain Reaction
RT-PCR: Real Time Polymerase Chain Reaction
