# Supplementary material for: Viral species richness and composition in young children with loose or watery stool in Ethiopia
Source: BMC Infect Dis. 2019 Jan 14;19:53. doi: 10.1186/s12879-019-3674-3 (PMC6332554; doi:10.1186/s12879-019-3674-3)
Supplement: Supplementary file 1 — Bioinformatics Pipeline. (PDF 29 kb) [file 12879_2019_3674_MOESM1_ESM.pdf]

**Bioinformatic analyses**

Overview: An in-house analysis pipeline was used to analyze sequence data. Raw data was first pre-processed by subtracting human and bacterial sequences, duplicate sequences, and low-quality reads. The reads are de novo assembled and contigs and singlet reads were aligned against a customized viral proteome database using BLASTx. Candidate viral hits were then compared to a non-virus non-redundant (nr) protein database to remove false positive viral hits.

Database compilation: To electronically subtract non-viral sequence the human reference genome sequence (hg38) and mRNA sequences were first concatenated. Bacterial nucleotide sequences were also extracted from NCBI nt fasta file (ftp://ftp.ncbi.nlm.nih.gov/blast/db/FASTA/, Oct. 20, 2017) based on NCBI taxonomy (ftp://ftp.ncbi.nih.gov/pub/taxonomy, Oct. 20, 2017). Human and bacterial nucleotide sequences were then compiled into bowtie2 (version 2.2.4) databases [26] for human and bacterial sequences subtraction. Two databases were constructed: 1) virus BLASTx database was compiled using NCBI virus reference proteome (ftp://ftp.ncbi.nih.gov/refseq/release/viral/, Oct. 20, 2017) to which was added viral proteins sequences from NCBI nr fasta file (based on annotation taxonomy in Virus Kindom); and 2) a non-virus nr (NVNR) database was compiled using non-viral protein sequences extracted from NCBI nr fasta file (based on annotation taxonomy excluding Virus Kindom). Repeats and low-complexity regions were masked using segmasker from blast+ suite (version 2.2.7)[27].

Preprocessing: Paired-end reads of 250 bp generated by MiSeq were debarcoded using vendor software from Illumina. Human host reads and bacterial reads are identified and removed by mapping the raw reads to human reference genome hg38 and bacterial genomes release 66 using bowtie2 in local search mode with other parameters set as default, requiring finding 60bp aligned segment with at most 2 mismatches and no gaps [26]. Reads were considered duplicates if 5bp to 55bp from 5 prime end are identical. One random copy of duplicates was kept. Duplicate sequences were replaced with sequence 'A' as a place holder; preserving the original order of the paired-end files for paired-end sequence assembly. A paired-end sequence record is removed if both paired reads are deleted duplicates. Low sequencing quality tails are trimmed using Phred quality score 20 as the threshold. Adaptor and primer sequences are trimmed using the default parameters of VecScreen using default parameters [27].

De novo assembly: We developed a strategy that integrates the sequential use of various de Bruijn graph (DBG) and overlap-layout-consensus assemblers (OLC) with a novel partitioned sub-assembly approach called ENSEMBLE [28].

Sequence reads were first analyzed using BLASTx (version 2.2.7) for translated protein sequence similarity to all viral protein sequences in GenBank's virus RefSeq database plus protein sequences taxonomically annotated as viral in GenBank's non-redundant database using E-value cutoff of 0.01. To remove background due to sequence misclassification these initial viral hits were then compared to all protein sequences in NR using the program DIAMOND (version 0.9.6) and retained only when the top hit was to a sequence annotated as viral. A threshold E score of $<10^{-10}$ was then used to ensure only reads with high levels of similarity to viral proteins were counted. Further analyses focused on eukaryotic viruses.

To align reads and contigs to reference viral genomes from GenBank and generate complete or partial genome sequences the Geneious R10 program was used.
