# Supplementary material for: Viral species richness and composition in young children with loose or watery stool in Ethiopia
Source: BMC Infect Dis. 2019 Jan 14;19:53. doi: 10.1186/s12879-019-3674-3 (PMC6332554; doi:10.1186/s12879-019-3674-3)
Supplement: Supplementary file 2 — Table S1. Prevalence of species/genotype by mBSFS-C stool consistency category. (PDF 58 kb) [file 12879_2019_3674_MOESM2_ESM.pdf]

Table S1: Prevalence of species/genotype by mBSFS-C stool consistency category

N pools (children)	Watery N=4 (29)	Loose N=8(79)	Smooth N=6(59)	Lumpy N=9(88)	Pellets N=2(14)	Overall N=29(269)
Adeno-associated dependoparvovirus A, AAV2	3 (75%)	0 (0%)	1 (16.7%)	0 (0%)	0 (0%)	4 (13.8%)
Aichivirus A	1 (25%)	2 (25%)	0 (0%)	1 (11.1%)	0 (0%)	4 (13.8%)
Anellovirus	3 (75%)	3 (37.5%)	2 (33.3%)	2 (22.2%)	0 (0%)	10 (34.5%)
Bufavirus 3	1 (25%)	0 (0%)	1 (16.7%)	0 (0%)	0 (0%)	2 (6.9%)
Cosavirus A	1 (25%)	4 (50%)	2 (33.3%)	2 (22.2%)	0 (0%)	9 (31%)
Cosavirus D	0 (0%)	1 (12.5%)	0 (0%)	0 (0%)	0 (0%)	1 (3.4%)
Cosavirus E	1 (25%)	0 (0%)	0 (0%)	0 (0%)	0 (0%)	1 (3.4%)
Cosavirus E/D	0 (0%)	0 (0%)	1 (16.7%)	1 (11.1%)	0 (0%)	2 (6.9%)
Enterovirus A, Coxsackievirus A14	0 (0%)	0 (0%)	0 (0%)	1 (11.1%)	0 (0%)	1 (3.4%)
Enterovirus A, Coxsackievirus A16	1 (25%)	2 (25%)	0 (0%)	0 (0%)	0 (0%)	3 (10.3%)
Enterovirus A, Coxsackievirus A6	0 (0%)	1 (12.5%)	0 (0%)	2 (22.2%)	0 (0%)	3 (10.3%)
Enterovirus B, Echovirus E14	0 (0%)	0 (0%)	1 (16.7%)	1 (11.1%)	0 (0%)	2 (6.9%)
Enterovirus B, Echovirus E16	1 (25%)	1 (12.5%)	0 (0%)	0 (0%)	0 (0%)	2 (6.9%)
Enterovirus B, Echovirus E18	0 (0%)	1 (12.5%)	1 (16.7%)	1 (11.1%)	0 (0%)	3 (10.3%)
Enterovirus B, Echovirus E19	1 (25%)	0 (0%)	1 (16.7%)	0 (0%)	0 (0%)	2 (6.9%)
Enterovirus B, Echovirus E27	0 (0%)	1 (12.5%)	0 (0%)	0 (0%)	0 (0%)	1 (3.4%)
Enterovirus B, Echovirus E6	0 (0%)	0 (0%)	1 (16.7%)	1 (11.1%)	0 (0%)	2 (6.9%)
Enterovirus C, Coxsackievirus A1	0 (0%)	1 (12.5%)	0 (0%)	0 (0%)	0 (0%)	1 (3.4%)
Enterovirus C, Coxsackievirus A13	0 (0%)	0 (0%)	0 (0%)	1 (11.1%)	0 (0%)	1 (3.4%)
Enterovirus C, Coxsackievirus A17	0 (0%)	1 (12.5%)	1 (16.7%)	1 (11.1%)	1 (50%)	4 (13.8%)
Enterovirus C, Coxsackievirus A20	0 (0%)	2 (25%)	2 (33.3%)	0 (0%)	0 (0%)	4 (13.8%)
Enterovirus C, Enterovirus C99	0 (0%)	0 (0%)	1 (16.7%)	1 (11.1%)	0 (0%)	2 (6.9%)
Hepatovirus A, Hepatovirus A_IB	0 (0%)	2 (25%)	2 (33.3%)	0 (0%)	0 (0%)	4 (13.8%)
Human bocaparvovirus 1, Human bocavirus 1	0 (0%)	0 (0%)	0 (0%)	1 (11.1%)	0 (0%)	1 (3.4%)
Human bocaparvovirus 1, Human bocavirus 3	1 (25%)	0 (0%)	0 (0%)	1 (11.1%)	0 (0%)	2 (6.9%)
Human bocaparvovirus 2, Human bocavirus 2	1 (25%)	2 (25%)	1 (16.7%)	1 (11.1%)	0 (0%)	5 (17.2%)
Human bocaparvovirus 2, Human bocavirus 4	1 (25%)	0 (0%)	0 (0%)	0 (0%)	1 (50%)	2 (6.9%)
Human mastadenovirus A	1 (25%)	1 (12.5%)	1 (16.7%)	2 (22.2%)	0 (0%)	5 (17.2%)
Human mastadenovirus C	1 (25%)	1 (12.5%)	1 (16.7%)	0 (0%)	0 (0%)	3 (10.3%)
Human mastadenovirus D	0 (0%)	2 (25%)	2 (33.3%)	0 (0%)	0 (0%)	4 (13.8%)
Human mastadenovirus F	1 (25%)	0 (0%)	0 (0%)	0 (0%)	0 (0%)	1 (3.4%)
Human picobirnavirus	0 (0%)	1 (12.5%)	0 (0%)	1 (11.1%)	0 (0%)	2 (6.9%)
Norwalk virus, Norovirus GI	0 (0%)	3 (37.5%)	1 (16.7%)	2 (22.2%)	0 (0%)	6 (20.7%)
Norwalk virus, Norovirus GII	2 (50%)	3 (37.5%)	0 (0%)	0 (0%)	0 (0%)	5 (17.2%)
Parechovirus A, Human parechovirus 1	1 (25%)	2 (25%)	2 (33.3%)	1 (11.1%)	1 (50%)	7 (24.1%)
Parechovirus A, Human parechovirus 17	0 (0%)	0 (0%)	0 (0%)	1 (11.1%)	0 (0%)	1 (3.4%)
Parechovirus A, Human parechovirus 4	1 (25%)	0 (0%)	0 (0%)	0 (0%)	0 (0%)	1 (3.4%)
Parechovirus A, Human parechovirus 5	0 (0%)	1 (12.5%)	0 (0%)	0 (0%)	0 (0%)	1 (3.4%)
Parechovirus A, Human parechovirus 6	0 (0%)	0 (0%)	0 (0%)	1 (11.1%)	0 (0%)	1 (3.4%)
Parechovirus A, Human parechovirus 8	0 (0%)	0 (0%)	0 (0%)	1 (11.1%)	0 (0%)	1 (3.4%)
Salivirus	2 (50%)	1 (12.5%)	1 (16.7%)	3 (33.3%)	1 (50%)	8 (27.6%)
Sapporo virus	0 (0%)	1 (12.5%)	0 (0%)	2 (22.2%)	0 (0%)	3 (10.3%)

Number of positive pools (%)

AAV2 = Adeno-associated virus 2
