# Supplementary figures and images for: Viral species richness and composition in young children with loose or watery stool in Ethiopia
Source: BMC Infect Dis. 2019 Jan 14;19:53. doi: 10.1186/s12879-019-3674-3 (PMC6332554; doi:10.1186/s12879-019-3674-3)

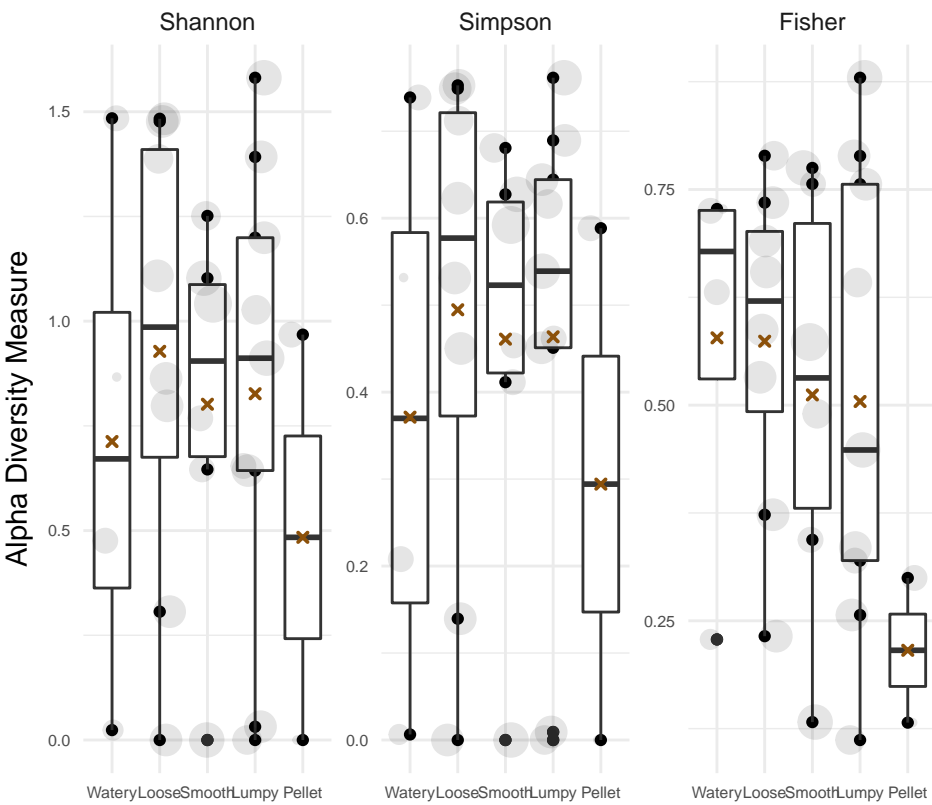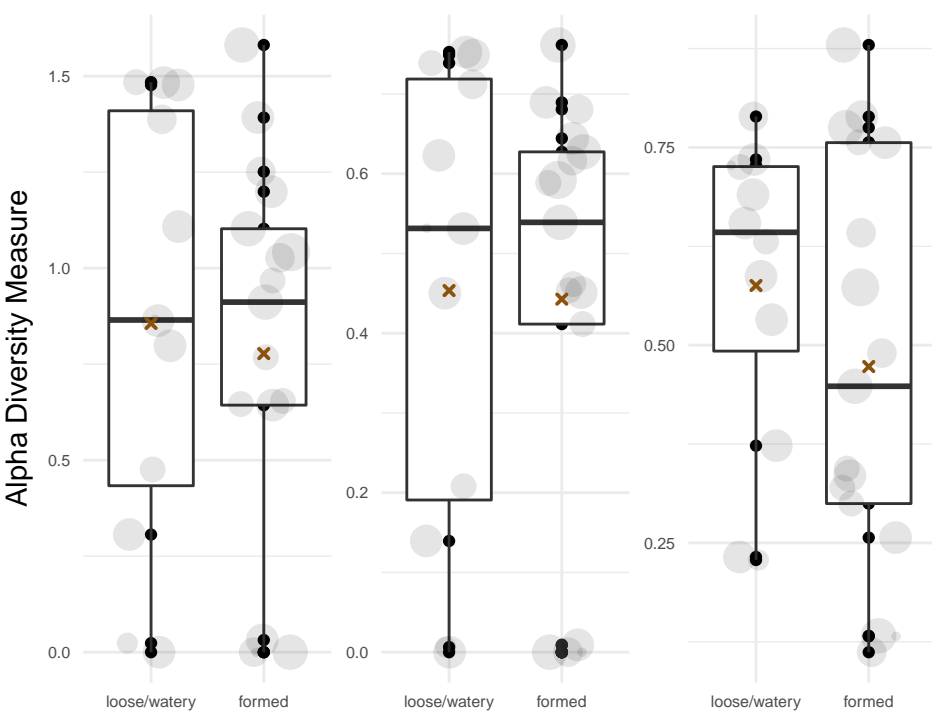

Supplement: Supplementary file 3 — Figure S1. Alpha Diversity according to mBSFS-C stool consistency category. (PDF 19 kb) [file 12879_2019_3674_MOESM3_ESM.pdf]
